# Development of a Clinical Prediction Model for Acute Kidney Injury Among In-Hospital Cardiac Arrest Patients During Intensive Care Unit Hospitalization

**DOI:** 10.31083/RCM47434

**Published:** 2026-04-08

**Authors:** Halidan Abudu, Ziquan Liu, Yanxiang Niu, Guowu Xu, Kang He, Jinrui Dong, Tayierjiang Tuerxun, Guansen Hua, Xiangyu Yan, Haojun Fan

**Affiliations:** ^1^School of Disaster and Emergency Medicine, Tianjin University, 300192 Tianjin, China; ^2^Department of Emergency Medicine, Tianjin Medical University General Hospital, 300052 Tianjin, China; ^3^Bioengineering College of Chongqing University, 400044 Chongqing, China; ^4^School of Medicine, Faculty of Medicine, Tianjin University, 300052 Tianjin, China; ^5^Urumqi Traditional Chinese Medicine Hospital, 830000 Urumqi, Xinjiang, China; ^6^Xiamen Peiyang BCI & Smart Health Innovation Research Institution, 361199 Xiamen, Fujian, China

**Keywords:** MIMIC-IV database, in-hospital cardiac arrest, cardiac arrest-associated acute kidney injury, prediction model

## Abstract

**Background:**

Acute kidney injury (AKI) is a significant cause of mortality among post-cardiac arrest patients. However, clinical prediction models for assessing AKI risk for in-hospital cardiac arrest (IHCA) patients remain limited. Thus, this retrospective study aimed to develop a nomogram that uses readily available clinical characteristics to predict the likelihood of AKI in this group of patients during intensive care unit (ICU) hospitalization.

**Methods:**

This study constructed a nomogram based on the Medical Information Mart for Intensive Care IV (MIMIC-IV) database and conducted variable selection through Least Absolute Shrinkage and Selection Operator (LASSO) regression, followed by univariate and multivariate logistic regression analyses on the selected variables. Model performance was evaluated by calculating sensitivity, specificity, and the Youden index, and by performing decision curve analysis (DCA), clinical impact curve (CIC), and receiver operating characteristic (ROC) curve analysis.

**Results:**

This study included 1427 cardiac arrest (CA) patients, who were randomly allocated into a training cohort (n = 999) and a validation cohort (n = 428). We identified five independent predictors for post-cardiac arrest AKI: weight (adjusted odds ratio (aOR): 1.016, 95% confidence interval (CI): 1.009–1.024), peripheral capillary oxygen saturation (SpO_2_) (aOR: 1.044, 95% CI: 1.026–1.063), sodium (aOR: 0.947, 95% CI: 0.919–0.975), Sequential Organ Failure Assessment (SOFA) score (aOR: 1.134, 95% CI: 1.083–1.190), and Oxford Acute Severity of Illness Score (OASIS) score (aOR: 1.080, 95% CI: 1.059–1.103). The model demonstrated strong performance, with area under the curve (AUC) values of 0.920 and 0.875 in the training and validation cohorts, respectively. Upon validation, the specificity, sensitivity, and Youden index for the model were 0.837, 0.781, and 0.618, respectively. The calibration curve indicated good agreement between predictions and observations. The DCA and CIC confirmed the clinical utility of the model.

**Conclusion:**

The developed prediction model exhibits high predictive performance for predicting AKI in IHCA patients.

## 1. Introduction

Despite significant improvements in preventive measures, cardiac arrest (CA) remains a serious public health issue [1]. In the United States, approximately 290,000 in-hospital cardiac arrests (IHCA) and 350,000 out-of-hospital cardiac arrests (OHCA) occur annually [2]. The incidence of IHCA ranges from approximately 1 to 17 per 1000 admissions, though the global incidence in adults has not been well characterized [3]. IHCA remains a relatively neglected condition compared to OHCA. A systematic review of 92 randomized CA clinical trials (1995–2014, ≥50 patients each) found that only 4 (4%) focused exclusively on IHCA patients [4]. The 2010 American Heart Association (AHA) Emergency Cardiovascular Care (ECC)Committee established goals to improve CA survival rates, aiming to double survival for both OHCA and IHCA by 2020. Current reported 30-day survival rates for patients with OHCA and IHCA are 17% and 24%, respectively [5], underscoring the necessity of focusing on both IHCA and OHCA to improve patient survival.

Acute kidney injury (AKI) occurs in approximately 50% of post-cardiac arrest patients [6] and has been associated with poor clinical outcomes in studies of cardiac intensive care unit patients [7]. AKI is a significant risk factor for unfavorable neurological outcomes and elevated mortality following CA [8, 9]. Previous research has indicated that severe AKI is associated with reduced hospital survival (48% versus 65%, *p* = 0.006) and reduced 12-month survival with favorable neurobehavioral outcomes (30% versus 53%, *p* < 0.001) [10].

In CA patients, early recognition of AKI is critical, as renal replacement therapy (RRT) becomes the only therapeutic option when there is severe progression of the disease. Wang *et al*. [11] established and validated a predictive model for early acute kidney injury after cardiac arrest resuscitation through retrospective collection of clinical cases. Similarly, Lin *et al*. [12] developed and validated a risk prediction model for AKI after cardiac arrest using clinical data. However, both studies share the same limitations: they are single-center retrospective studies with relatively small sample sizes and limited included variables. Therefore, developing robust risk prediction models for cardiac arrest-associated acute kidney injury (CA-AKI) is necessary. Such models would facilitate early and accurate detection of CA-AKI, enabling timely intervention before severe progression occurs.

## 2. Materials and Methods

### 2.1 Research Data Source

We acquired the study data from a comprehensive critical care database named Medical Information Mart for Intensive Care IV (MIMIC-IV), which is equipped for multi-parameter intelligent monitoring. MIMIC-IV is an openly available database containing intensive care information from more than forty thousand patients from 2008 to 2022. This database was established following ethical and regulatory guidelines, having received approval from both the Massachusetts Institute of Technology (MIT) and the Beth Israel Deaconess Medical Center (BIDMC) [13]. The researcher successfully completed the required Human Subject Research Course (Certification Record: 58084498) and secured official permission to use the database.

The MIMIC-IV database used in this study was approved by the Institutional Review Boards (IRB) of both the Massachusetts Institute of Technology and the Beth Israel Deaconess Medical Center. Since the database contains no protected health information and all patient data are de-identified, individual patient consent was not required for this research.

### 2.2 Patient Inclusion Criteria

**Fig. 1.**
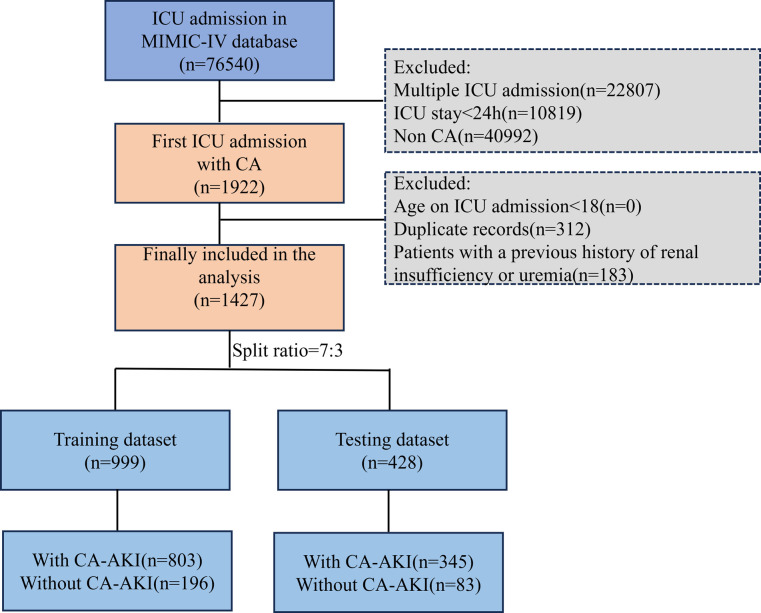
**Graphical depiction of the participant inclusion procedure in the study**. MIMIC-IV, medical information mart for intensive care IV; ICU, intensive care unit; CA, cardiac arrest; CA-AKI, cardiac arrest-associated acute kidney injury.

This study retrospectively analyzed intensive care unit (ICU) inpatient records from the Massachusetts Institute of Technology Beth Israel Deaconess Medical Center between 2008 and 2022. Eligible patients satisfied the following set of criteria: (1) age ≥18 years, (2) A confirmed CA diagnosis using the International Classification of Diseases (ICD) codes leading to their first ICU admission. Excluded criteria: (1) Multiple ICU admissions; (2) ICU time less than 24 h; (3) Non-CA patients; (4) duplicate records; (5) patients with a previous history of renal insufficiency or uremia. In the final analysis, a total of 1427 cases of CA patients were chosen and randomly distributed in a 7:3 proportion to the training dataset and the testing dataset, respectively (Fig. 1).

### 2.3 Outcome and Predictions

We sought to create an easily applicable clinical prediction tool for estimating the risk of AKI in CA patients admitted to the ICU. The primary outcome was ICU-acquired AKI, for which we used the first ICU serum creatinine value within 24 hours as the surrogate baseline to: (1) compute the KDIGO-defined ΔScr, and (2) eGFR values were obtained based on sex, age, and serum creatinine.

LASSO regression was selected for variable screening due to its computational efficiency, ability to generate sparse solutions, and robustness against multicollinearity and overfitting-advantages highlighted in comparative analyses with methods such as Horseshoe [14], which, despite theoretical optimality, lacks LASSO’s practical applicability in large-scale datasets and interpretable model construction.

The study incorporated a dataset comprising ICU-admitted patients, for whom demographic characteristics were meticulously documented. On the first day of the ICU admission, a comprehensive evaluation of relevant factors was conducted using the database. This evaluation encompassed demographic characteristics (age, sex, weight), vital signs (heart rate, respiratory rate, blood pressure), oxygen saturation, blood checks (white blood cells, platelet, hemoglobin), blood gas analysis (pH, PaCO_2_, lactate), biochemical markers (Creatinine, Lactate Dehydrogenase [LDH]), electrolytes (sodium, potassium), common severity scores (Sequential Organ Failure Assessment [SOFA], Systemic Inflammatory Response Syndrome [SIRS], Simplified Acute Physiology Score II [SAPS II]), AKI stage, and underlying diseases (hypertension, diabetes).

### 2.4 Statistical Analysis

We addressed missing values in the dataset using the multivariate imputation by chained equations (MICE) package (version 3.14.0; Stef van Buuren, University of Utrecht, Utrecht, Netherlands) in R (version 4.3.1; R Foundation for Statistical Computing, Vienna, Austria). (**Supplementary Table 1**). We first analyzed the distribution and proportion of missing data with the md.pattern() function and VIM visualization tools to perform imputation strategies. We applied multiple imputation via the MICE algorithm, selecting Predictive Mean Matching (PMM) for continuous variables (e.g., age, weight), logistic regression (logreg) for binary variables (ventilation type, AKI occurrence), and polynomial regression (polyreg) for categorical variables (race, gender). Imputation parameters were set to m = 5 imputations, maxit = 50 iterations, and a random seed of 500 for reproducibility. We assessed convergence and imputation quality using diagnostic plots and combined results following Rubin’s rules to account for imputation uncertainty, ensuring the rigor and validity of our statistical inferences. The enrolled patients were randomly partitioned into a training set and an internal validation set at a 7:3 ratio via the random number method. In the multicenter MIMIC-IV database, LASSO regression was conducted for variable selection. The logistic regression model was used to construct the final prediction model. The receiver operating characteristic (ROC) curve of the prediction model was plotted, and the area under the curve (AUC) was calculated to evaluate the predictive ability of the model. The calibration curve was used to evaluate the consistency between the actual probability and the predicted probability, and the decision curve analysis (DCA) was constructed to evaluate the net clinical benefit under different threshold probabilities. Statistical significance was set at *p* < 0.05. Using Eqns. 1,2,3, the specificity, sensitivity, and Youden index of the prediction model were calculated. R 4.3.1 software was used for baseline data statistics and related data analysis.

(1) $$\text{ Specificity }=\frac{T\,N}{F\,P+T\,N}$$

(2) $$\text{ Sensitivity }=\frac{T\,P}{T\,P+F\,N}$$

(3) Youden index =( Sensitivity + Specificity )-1

In the three formulas, TN, FP, TP, and FN denote True Negative, False Positive, True Positive, and False Negative, respectively.

## 3. Results

### 3.1 Basic Characteristics of Patients

Data from a total of 76,540 patients were retrieved through the MIMIC-IV database during the study period. After exclusion, 1427 patients were included in the final analysis. Among them, 999 patients were split into the training dataset and 428 into the testing dataset (Fig. 1).

Compared to patients without CA-AKI, those CA-AKI had an average age of 66.97 ± 16.79 versus 66.58 ± 18.42; the proportion of males in both groups was relatively high, with 60% in the CA-AKI group versus 56% in the without CA-AKI group; the average weight of the patients in the two groups was 84.26 ± 24.16 kg versus 77.42 ± 19.78 kg (*p* < 0.001); the serum creatinine levels were 1.80 ± 1.79 mg/dL for the CA-AKI group versus 1.37 ± 1.36 mg/dL for the without CA-AKI group (*p* < 0.001); the SOFA scores were 9.23 ± 4.36 for the CA-AKI group versus 5.41 ± 4.06 for the without CA-AKI group (*p* < 0.001); when comparing the two groups, a majority of CA-AKI patients were in stage 3 of AKI, with 43% versus 13% (*p* < 0.001); both groups had varying degrees of underlying diseases, with hypertension affecting 42% of the CA-AKI group versus 40% of the without CA-AKI group, and diabetes affecting 31% of the CA-AKI group versus 25% of the without CA-AKI group (Table 1).

**Table 1.** **The baseline characteristics of patients**.

Variable			Without CA-AKI (n = 279^1^)	CA-AKI (n = 1148^1^)	*p*-value^2^
Demographic characteristics					
	Age (year)		66.58 (18.42)	66.97 (16.79)	0.746
	Gender				0.174
		Female (%)	123 (44%)	454 (40%)	
		Male (%)	156 (56%)	694 (60%)	
	Weight (Kg)		77.42 (19.78)	84.26 (24.16)	<0.001
Vital indicators					
	Heart rate (beats/min)		91.75 (24.69)	90.81 (22.74)	0.566
	Respiratory rate (breaths/min)		19.51 (6.80)	20.03 (6.89)	0.254
	Systolic blood pressure (mmHg)		119.96 (29.10)	132.23 (14.9)	0.254
	Diastolic blood pressure (mmHg)		68.59 (20.69)	69.94 (20.56)	0.328
	Mean blood pressure (mmHg)		80.71 (21.07)	82.78 (21.50)	0.143
	SpO_2_ (%)		94.34 (10.21)	96.20 (7.25)	0.004
	Temperature (°C)		36.56 (5.10)	36.67 (6.73)	0.390
Laboratory indicators					
	White blood cells (K/uL)		13.04 (8.85)	13.87 (8.46)	0.155
	Platelet (K/uL)		206.23 (89.05)	214.76 (112.05)	0.175
	Hemoglobin (g/dL)		11.53 (2.58)	11.54 (2.52)	0.922
	Sodium (mEq/L)		139.57 (5.76)	138.64 (5.51)	0.015
	Potassium (mEq/L)		4.31 (0.81)	4.35 (0.92)	0.448
	Glucose (mg/dL)		177.49 (107.80)	182.30 (102.64)	0.501
	pH		7.28 (0.17)	7.30 (0.14)	0.239
	PaCO_2_ (mmHg)		45.38 (14.85)	44.53 (15.15)	0.392
	Lactate (mmol/L)		3.82 (3.54)	3.68 (3.06)	0.531
	Anion gap (mEq/L)		16.92 (5.74)	17.18 (5.23)	0.490
	Total carbon dioxide (mEq/L)		22.72 (6.94)	22.59 (6.18)	0.769
	Urea nitrogen (mg/dL)		28.00 (25.93)	30.68 (22.73)	0.113
	Creatinine (mg/dL)		1.37 (1.36)	1.80 (1.79)	<0.001
	LDH (IU/L)		714.01 (661.01)	853.80 (796.8)	0.148
	Magnesium (mEq/L)		2.03 (0.40)	2.05 (0.47)	0.457
Disease severity scoring system					
	SOFA		5.41 (4.06)	9.23 (4.36)	<0.001
	APS III		50.53 (26.01)	73.76 (30.20)	<0.001
	SIRS		2.31 (1.13)	2.79 (1.01)	<0.001
	SAPS II		37.06 (15.29)	49.47 (16.77)	<0.001
	OASIS		31.67 (9.97)	40.93 (9.57)	<0.001
Mechanical ventilation					
	Invasive mechanical ventilation		1.29 (0.75)	2.25 (2.77)	<0.001
AKI stage					<0.001
	Stage 1		0 (0%)	194 (17%)	
	Stage 2		0 (0%)	459 (40%)	
	Stage 3		0 (0%)	495 (43%)	
Underlying diseases					
	Hypertension		112 (40%)	482 (42%)	0.589
	Diabetes		70 (25%)	357 (31%)	0.049

^1^Mean (SD); n/N (%). ^2^Welch two-sample *t*-test; Fisher’s exact test; Fisher’s Exact Test for Count Data with simulated *p*-value (based on 2000 replicates). LDH, Lactate Dehydrogenase; SOFA, Sequential Organ Failure Assessment; APS III, Acute Physiology Score III; SIRS, Systemic Inflammatory Response Syndrome; SAPS II, Simplified Acute Physiology Score II; OASIS, Oxford Acute Severity of Illness Score; AKI, Acute Kidney Injury.

### 3.2 Model Development

**Fig. 2.**
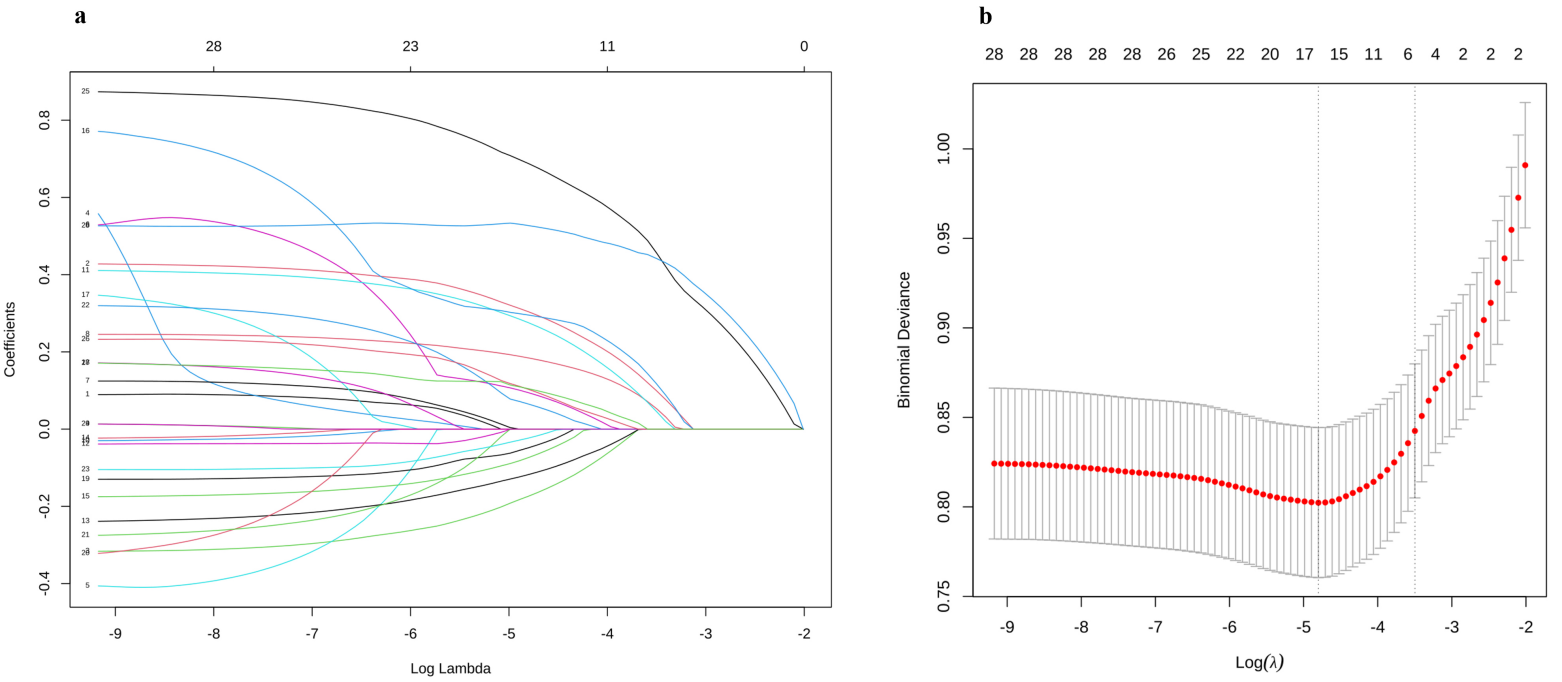
**Schematic diagram of clinical feature selection**. (a) The profile of LASSO estimates for predictive variables is illustrated, where the left vertical line marks the location of the optimal lambda, and the right vertical line indicates the position that is one standard error distant from the optimal lambda. (b) The graphic depicts the cross-validation curve for LASSO regression. The left dashed vertical line indicates both the count of features and the optimal logarithmic (lambda) value corresponding to the lowest mean squared error. λ, lambda.

**Fig. 3.**
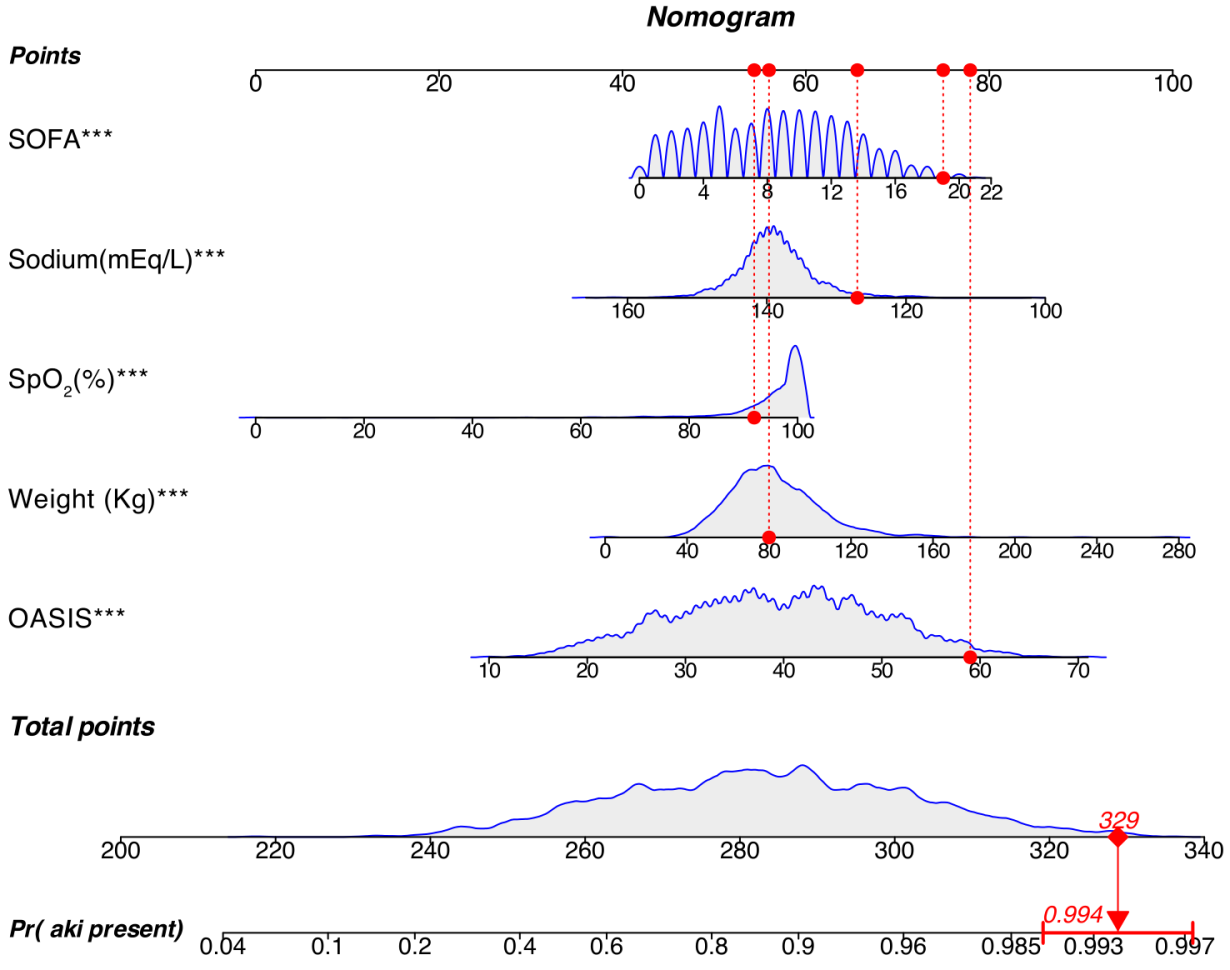
**Nomogram developed for the prediction of AKI incidence in patients with IHCA**. The nomogram assigns scores to each variable, enabling the assessment of AKI probability by summing the scores associated with the patient’s specific values. The red dot represents a selected positive patient within the cohort, with a cumulative score of 329 (*p* = 0.994), indicating a 99.4% probability of AKI occurrence for this patient. SOFA, Sequential Organ Failure Assessment; OASIS, Oxford Acute Severity of Illness Score; AKI, acute kidney injury; IHCA, in-hospital cardiac arrest. ***p < 0.001.

The study included 1427 patients, and after LASSO regression, 28 variables were selected and underwent logistic regression analysis (Fig. 2 and Table 2). We have included the variables that are statistically significant in the univariate logistic regression. These features were put through an additional binary multivariable logistic regression analysis and created a nomogram. This analysis led to the identification of five independent predictors for patients with CA-AKI, which include: weight (Adjusted Odds Ratio [aOR]: 1.016, 95% CI: 1.009–1.024), *p* < 0.001), SpO_2_ (aOR: 1.044, 95% CI: 1.026–1.063, *p* < 0.001), sodium (aOR: 0.947, 95% CI: 0.919–0.975, *p* < 0.001), SOFA (aOR: 1.134, 95% CI: 1.083–1.190, *p* < 0.001) and Oxford Acute Severity of Illness Score (OASIS) (aOR: 1.080, 95% CI: 1.059–1.103, *p* < 0.001) (**Supplementary Table 2**, Table 3 and **Supplementary Fig. 1**). We created the nomogram based on these findings (Fig. 3).

**Table 2.** **Univariate logistic regression analysis of independent predictors associated with AKI occurrence among IHCA patients**.

Variable	OR^1^ (95% CI^1^)	*p*-value^2^
Age	1.001 (0.994–1.009)	0.732
Weight (Kg)	1.015 (1.008–1.021)	<0.001
Heart rate (beats/min)	0.998 (0.993–1.004)	0.546
Systolic blood pressure (mmHg)	1.002 (1.000–1.007)	0.272
Diastolic blood pressure (mmHg)	1.003 (0.997–1.010)	0.324
Mean blood pressure (mmHg)	1.005 (0.998–1.011)	0.146
Respiratory rate (breaths/min)	1.011 (0.992–1.032)	0.253
SpO_2_ (%)	1.024 (1.010–1.040)	0.001
Temperature (°C)	0.992 (0.968–1.012)	0.454
White blood cells (K/uL)	1.013 (0.996–1.031)	0.131
Platelet (K/uL)	1.001 (1.000–1.002)	0.229
Hemoglobin (g/dL)	1.003 (0.952–1.056)	0.921
Sodium (mEq/L)	0.970 (0.946–0.993)	0.011
Potassium (mEq/L)	1.055 (0.912–1.227)	0.479
Glucose (mg/dL)	1.000 (0.999–1.002)	0.482
pH	1.794 (0.746–4.246)	0.190
PaCO_2_ (mmHg)	0.996 (0.988–1.005)	0.402
Lactate (mmol/L)	0.986 (0.948–1.028)	0.496
Anion gap (mEq/L)	1.009 (0.985–1.035)	0.462
Total carbon dioxide (mEq/L)	0.997 (0.976–1.018)	0.753
Urea nitrogen (mg/dL)	1.006 (0.999–1.012)	0.074
Creatinine (mg/dL)	1.255 (1.119–1.434)	<0.001
LDH (IU/L)	1.000 (1.000–1.000)	0.181
Magnesium (mEq/L)	1.108 (0.830–1.499)	0.493
SOFA	1.242 (1.199–1.289)	<0.001
APS III	1.031 (1.026–1.037)	<0.001
SAPS II	1.054 (1.044–1.064)	<0.001
OASIS	1.106 (1.089–1.124)	<0.001

^1^OR, Odds Ratio; CI, Confidence Interval. ^2^False discovery rate correction for multiple testing. SOFA, Sequential Organ Failure Assessment; IHCA, in-hospital cardiac arrest.

**Table 3.** **Using a backward method, we performed a multivariable logistic regression analysis to find independent predictors related to AKI in IHCA patients**.

Variable	AOR^1^ (95% CI^1^)	*p*-value^2^
Weight (Kg)	1.016 (1.009–1.024)	<0.001
SpO_2_ (%)	1.044 (1.026–1.063)	<0.001
Sodium (mEq/L)	0.947 (0.919–0.975)	<0.001
SOFA	1.134 (1.083–1.190)	<0.001
OASIS	1.080 (1.059–1.103)	<0.001

^1^AOR, Adjusted Odds Ratio; CI, Confidence Interval. ^2^False discovery rate correction for multiple testing.

### 3.3 Evaluation of Model Efficacy

**Fig. 4.**
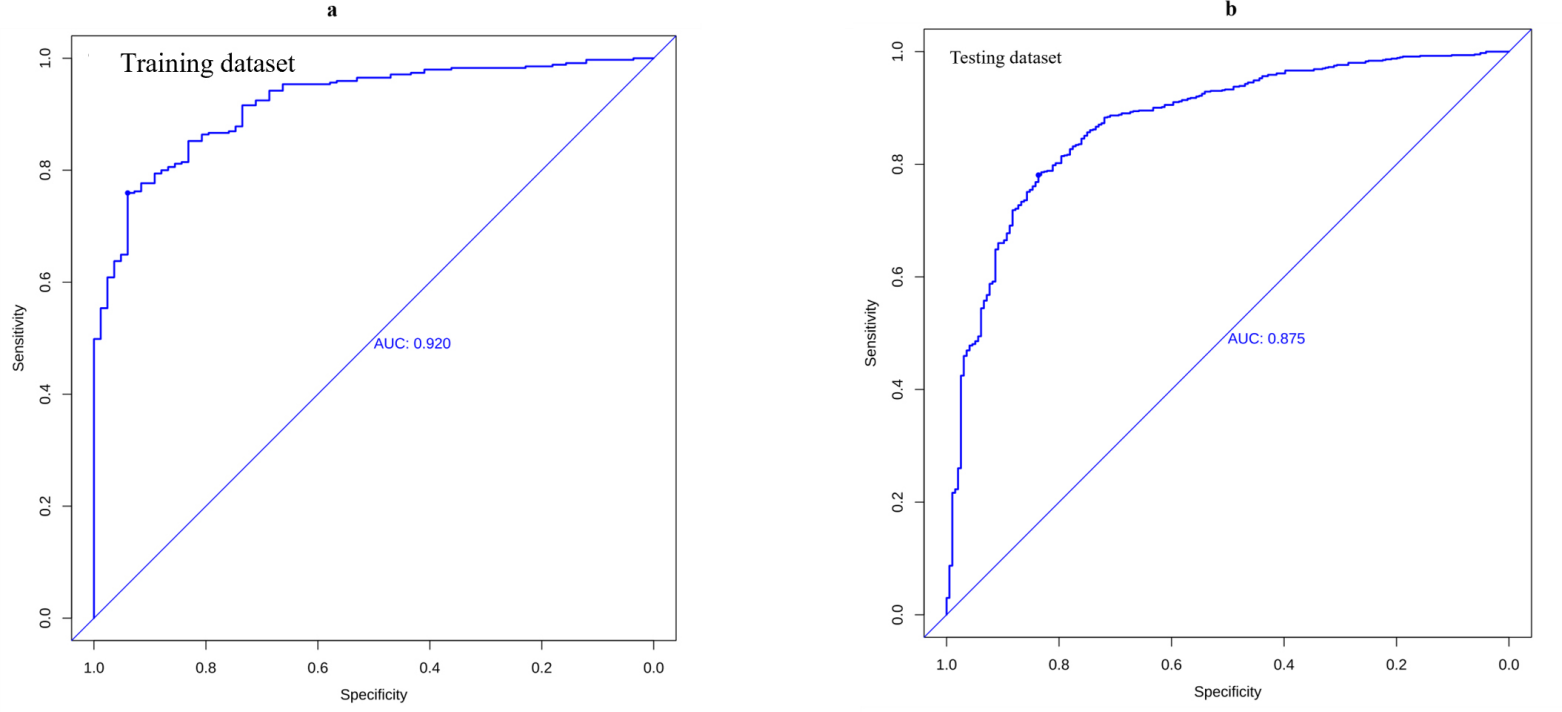
**ROC analyses**. The model showed good discrimination for CA-AKI, with areas under the curves (AUCs) of 0.920 in the training dataset (a) and 0.875 in the testing dataset (b). CI, confidence interval; ROC, receiver operating; AUC, area under the curve.

**Fig. 5.**
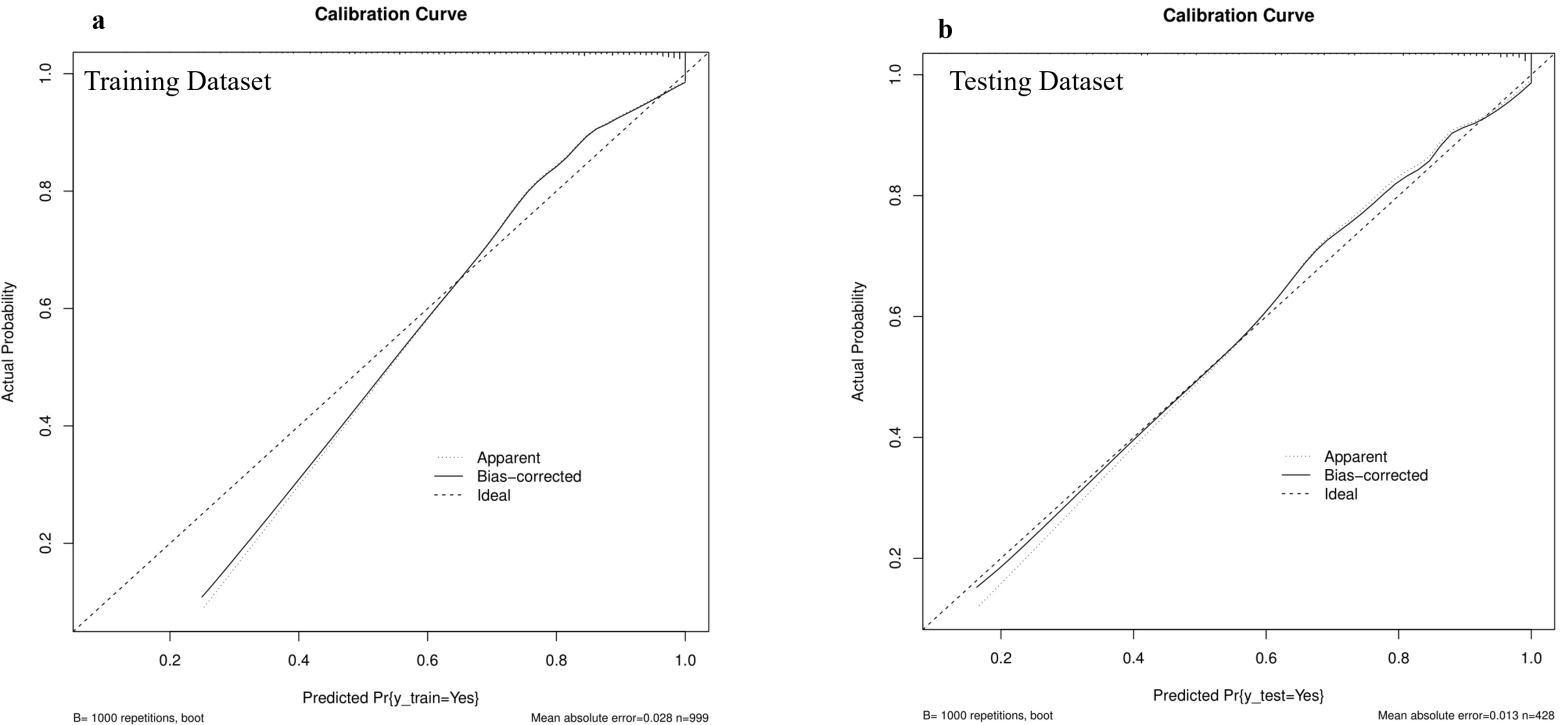
**Calibration plots in the training and testing datasets**. Calibration plots showed high accuracy in predicting absolute risk, as evidenced by the results of (a) the training dataset and (b) the testing dataset.

ROC analysis assessed model discrimination using AUC calculations. The prediction model showed strong discriminative ability for CA-AKI in the training data and testing dataset with AUC values of 0.920 and 0.875, respectively (Fig. 4a,b). The training dataset showed specificity, sensitivity, and Youden index values of 0.940, 0.757, and 0.697. The test dataset showed corresponding values of 0.837, 0.781, and 0.618. Strong concordance was observed between predicted and actual probabilities in both the training and testing sets (Fig. 5a,b).

### 3.4 Clinical Utility Validation

**Fig. 6.**
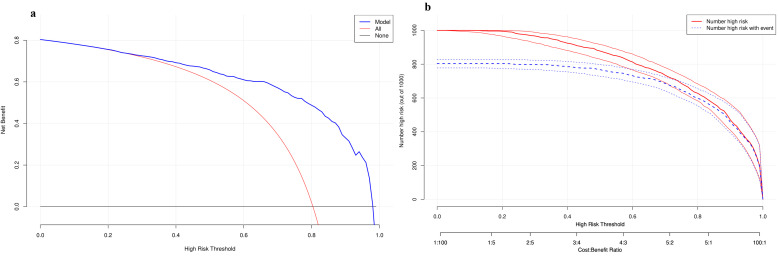
**Decision curve analysis and clinical impact curve in testing in training dataset**. (a) Decision curve analysis. (b) Clinical impact curve.

DCA and clinical impact curves (CIC) were used to assess the clinical usefulness. The DCA demonstrated that the predictive model provided a net benefit across nearly all threshold probabilities, confirming its clinical utility across a range of high-risk thresholds. At lower risk thresholds (e.g., <0.4), the model achieved a higher net benefit compared to the “treat-all” strategy (red line), indicating its ability to avoid unnecessary interventions in low-risk individuals. As the risk threshold increased, the model maintained superior net benefit until approaching the highest thresholds (near 1.0), where the “treat-none” strategy (gray line) became equivalent. This highlights the model’s robustness in guiding cost-effective clinical decisions across diverse risk tolerance levels (Fig. 6a). The CIC illustrates the practical consequences of applying the model in a clinical setting, showing the trade-off between the number of patients identified for intervention and the number of true events captured. The solid red line (“Number high risk”) represents the number of individuals (out of 1000) who would be classified as high-risk at a given threshold. The dashed blue line (“Number high risk with event”) shows the number of true positive cases within that high-risk group (Fig. 6b).

## 4. Discussion

Using MIMIC-IV data, we developed a well-calibrated CA-AKI prediction model with routinely available clinical variables.

The model incorporated five accessible predictors (weight, SpO_2_, sodium, SOFA, OASIS) and demonstrated clinical utility via DCA/CIC analysis, enabling early high-risk CA-AKI identification and improved clinical decisions.

Demographic indicators are important variables in predictive models. This study found that body weight is positively correlated with the occurrence of CA-AKI. Overweight, as the second major risk factor for cardiovascular diseases [15], can significantly increase the risk of conditions such as hypertension, acute coronary syndrome (ACS), diabetes, and dyslipidemia, all of which are important predisposing factors for CA [16]. A recent study revealed that among patients with ACS, the overall incidence of sudden cardiac arrest (SCA) is as high as 17.5% [17]. Obesity can affect renal function through obesity-related glomerulopathy (ORG) and alterations in renal hemodynamics. Activation of the renin-angiotensin-aldosterone system (RAAS), hypertension, renal lipotoxicity mediated by adipokines, and activation of the nucleotide-binding oligomerization domain-like receptor pyrin domain-containing 3 (NLRP3) inflammasome can all exacerbate renal damage [18]. Therefore, an increase in body weight not only elevates the risk of CA but also promotes the occurrence of AKI through multiple mechanisms. A study conducted at Santo Bortolo Hospital in Italy included 142 patients with CA-AKI. The results showed that the body mass index (BMI) of these patients was 25.2 (23.8–27.7), falling within the overweight range [19]. This result is consistent with the conclusion of our study, which indicates that body weight is positively correlated with the occurrence of CA-AKI.

Vital signs are one of the most commonly used and important indicators in predictive models. A growing body of animal experimental and clinical data suggests that immediate inhalation of 100% oxygen CA is prone to causing hyperoxemia. Elevated oxygen partial pressure can increase the production of oxygen-free radicals, exacerbate reperfusion injury, and be associated with unfavorable neurological outcomes. Although some studies have adjusted the target SpO_2_ for patients with OHCA after return of spontaneous circulation (ROSC) from 98–100% down to 90–94%, this adjustment has not significantly improved the discharge survival rate [20]. Excessive oxygen administration can also disrupt the balance between the production and clearance of reactive oxygen species within the kidneys, promoting cell apoptosis, inflammatory cascades, and renal tissue fibrosis, thus providing a pathological basis for AKI [21]. A recent study found that in comatose OHCA patients, those who simultaneously received a lower mean arterial pressure and a lenient oxygen delivery strategy had a significantly higher risk of AKI [22]. In this study, we observed a negative correlation between SpO_2_ levels and the incidence of CA-AKI, indicating that precise control of oxygenation targets in the management of vital signs for IHCA patients may be a crucial step in reducing renal injury and improving long-term prognosis.

During the construction of predictive models, laboratory indicators are often used as surrogate markers to indirectly reflect clinical outcomes. Sodium metabolism disorders are extremely common in the ICU, yet they are rarely incorporated into the prognostic assessment of CA. Current data show that among comatose CA patients who have achieved ROSC, approximately 20% already exhibit hyponatremia upon admission to the ICU, while hypernatremia is rare. One study has demonstrated that even after adjustments, hyponatremia remains associated with a reduced probability of attaining a favorable functional outcome at 180 days [23]. The underlying mechanism for these findings is that sodium ions are involved in the electrical conduction of myocardial cells. Severe hyponatremia can induce paroxysmal bradyarrhythmias, which may subsequently lead to pulseless electrical activity or even CA [24]. The kidneys are a key organ for regulating water-electrolyte balance. In clinical practice, AKI often coexists with sodium metabolism disorders. When renal function is impaired, both urine dilution and concentration functions may be affected. Abnormalities in urine dilution function, coupled with excessive water intake by the patient, can result in an excess of water in the body, leading to dilutional hyponatremia. Conversely, if the urine concentration function is impaired and the patient has insufficient water intake, excessive water loss from the body can occur, resulting in hypernatremia. These conditions underscore the critical role of the kidneys in regulating water and sodium balance, particularly during AKI, when impaired renal function significantly affects the metabolic equilibrium of sodium and water. Although existing literature has reported a linear relationship between the coefficient of variation in serum sodium levels and the risk of AKI, the causal relationship between the two remains unclear [25]. In cases of AKI, the incidence of sodium metabolism disorders ranges from 22.5% to 24.6%, and patients with such disorders face a significantly higher risk of mortality [26]. Marahrens *et al*. [27] found that serum sodium levels were associated with the risk of in-hospital mortality in AKI patients, with hypernatremic patients exhibiting a higher mortality risk. Additionally, another study also indicated that both hyponatremia and hypernatremia were associated with poor outcomes in AKI patients [28]. In this study, we observed a negative correlation between serum sodium levels and the risk of CA-AKI. In summary, sodium metabolism disorders are not only related to the occurrence of AKI but also closely associated with the prognosis of CA patients. Therefore, for patients with sodium metabolism disorders, close monitoring of their cardiac rhythm and renal function is crucial to promptly identify and manage potential risks.

In clinical practice, various scoring systems are frequently incorporated as predictive variables into clinical prediction models. The APACHE II score is the most widely used disease severity scoring system in ICUs worldwide [29]. However, it requires the collection of 12 physiological and laboratory indicators, along with 2 disease-related variables [30], making data acquisition time-consuming and not conducive to early, rapid assessment. The SOFA score, on the other hand, is used to quantify organ dysfunction. Studies have shown that the SOFA score is an independent risk factor for mortality in elderly ICU patients [31] and can objectively assess the severity of the post-cardiac arrest syndrome (PCAS) [32]. When combined with blood lactate levels, the SOFA score demonstrates improved predictive performance for the discharge survival rate of PCAS patients [33]. A study conducted by the University of Science and Technology of China, which included 347 patients with CA (197 cases in the CA-AKI group and 150 cases in the no CA-AKI group), revealed that although there was no statistically significant difference in the SOFA scores between the AKI group and the no-AKI group (11.294 ± 3.400 versus 10.840 ± 3.595), the mean score in the AKI group was slightly higher than that in the no-AKI group [34]. In this study, we also found a positive correlation between the SOFA score and the risk of CA-AKI. The OASIS score, which does not require laboratory or imaging tests, is simple and easy to use and has been proven to effectively discriminate the condition and prognosis of ICU patients [35]. A multicenter prospective study compared the predictive abilities of OASIS, APACHE II, SAPS II, and SOFA for 28-day mortality in ICU patients with acute kidney injury (ICU-AKI). The results indicated that OASIS performed the best (an OASIS score >33 suggested a poor short-term prognosis) [36]. In summary, the predictive value of SOFA and OASIS in patients with AKI, CA, and those in the ICU has been validated. However, their predictive performance for CA-AKI remains unclear. This study confirmed that both scores were positively correlated with the risk of CA-AKI. Ideally, a “gold standard” should be used for clinical diagnosis. However, when CA patients are admitted to the ICU, they often lose consciousness and are subject to numerous objective constraints, making it difficult to immediately conduct gold standard examinations. Under such circumstances, the rational application of the aforementioned scoring systems can assist clinicians in rapidly assessing the patient’s condition and predicting the prognosis.

This prediction model has several advantages in the management and prognosis of patients with CA. First, it is helpful for the early detection of CA-AKI patients. One study have found that whether the condition of patients with severe AKI can improve and recover early has a significant impact on their survival rates, hospitalization rates, and other outcomes [37]. The remission time of AKI is related to the long-term prognosis of patients. Research has shown that if AKI resolved within 7 days, the 1-year survival rate exceeded 90%. In contrast, the 1-year survival rate after discharge was only 77% [38]. Therefore, early identification of CA-AKI through prediction models is crucial for promptly adopting intervention measures. Early intervention can prevent adverse neurological outcomes, reduce disability rates, and improve survival rates. Second, it is helpful for the rational allocation of clinical resources. This prediction model aids in the rational allocation of clinical resources, such as dialysis machines and intensive care unit beds, thereby enabling more efficient use of limited medical resources and improving patient outcomes. Moreover, continuous refinement of the model based on real-world feedback can further enhance its performance and clinical utility.

### Limitations

This study has certain limitations. Despite the large sample size of the MIMIC-IV database, there may still be implicit data entry errors and selection bias; therefore, we have conducted rigorous cleaning and processing of extreme values and missing data. Second, since this study is retrospective, further prospective studies are needed to enhance the clinical practicality and effectiveness of the predictive model we have constructed. Third, in this study, we employed LASSO regression for variable screening; however, it should be noted that this method may have the limitation of excessively compressing variable coefficients. Fourth, it is important to emphasize that the validation of the nomogram is confined to internal assessment, which highlights the necessity of conducting external validation to determine its performance and reliability.

## 5. Conclusion

This research has identified a set of independent predictors closely linked to the occurrence of AKI in patients with IHCA who were admitted to the ICU. These predictors encompassed a range of factors, specifically weight, SpO_2_, sodium, SOFA, and OASIS. The prediction model that was developed based on these predictors demonstrated promising predictive results, accurately forecasting the likelihood of the development of AKI. Moreover, it exhibited significant clinical utility in the risk evaluation of AKI among ICU-admitted IHCA patients, aiding the medical staff in making more informed treatment decisions.

Future studies should not only deploy this model across diverse clinical environments but also rigorously validate its efficacy in a broader spectrum of settings to ensure its generalizability and widespread applicability. It would also be worthwhile to investigate the feasibility of integrating this model into electronic health records (EHR) or real-time clinical decision-support systems, which could offer a practical and forward-looking perspective for future research endeavors.

## Availability of Data and Materials

The datasets supporting the findings of this study are available from the corresponding author upon reasonable request.

## Supplementary Material

Supplementary material associated with this article can be found, in the online version, at https://doi.org/10.31083/RCM47434.
