# Supplementary material for: Development of a Clinical Prediction Model for Acute Kidney Injury Among In-Hospital Cardiac Arrest Patients During Intensive Care Unit Hospitalization
Source: Rev Cardiovasc Med. 2026 Apr 8;27(4):47434. doi: 10.31083/RCM47434 (PMC13155988; doi:10.31083/RCM47434)
Supplement: Supplementary file 1 [file 2153-8174-27-4-47434-s1.zip › Supplementary Material.docx]

**Supplementary Table 1.** **Variable Characteristics: Missingness, Percentages, and Ranges**

| **Variable** | **Min Value** | **Max Value** | **Missing Count** | **Missing Percentage (%)** |
| --- | --- | --- | --- | --- |
| LDH(IU/L) | 94 | 1998 | 457 | 32.03 |
| Systolic Blood Pressure(mmHg) | 18 | 314 | 451 | 31.6 |
| Diastolic Blood Pressure(mmHg) | 39 | 150 | 451 | 31.6 |
| Mean Blood Pressure(mmHg) | 38 | 200 | 442 | 30.97 |
| AKI Stage | 1 | 3 | 279 | 19.55 |
| Temperature (℃) | 30 | 40.28 | 165 | 11.56 |
| Invasive Mechanical Ventilation |  |  | 165 | 11.56 |
| PaCO_2_(mmHg) | 11 | 145 | 144 | 10.09 |
| Total Carbon Dioxide(mEq/L) | 5 | 45 | 144 | 10.09 |
| Lactate(mmol/L) | 0.5 | 22 | 140 | 9.81 |
| pH | 6.68 | 7.78 | 128 | 8.97 |
| Magnesium(mEq/L) | 0.7 | 6.4 | 45 | 3.15 |
| White Blood Cells(K/ul) | 0.1 | 102.3 | 44 | 3.08 |
| Hemoglobin(g/dL) | 2.8 | 20 | 44 | 3.08 |
| Anion Gap(mEq/L) | 4 | 45 | 43 | 3.01 |
| Potassium(mEq/L) | 1.6 | 9.1 | 42 | 2.94 |
| Glucose(mg/dL) | 15 | 946 | 42 | 2.94 |
| Sodium(mEq/L) | 102 | 166 | 41 | 2.87 |
| Creatinine(mg/dL) | 0.3 | 23.1 | 41 | 2.87 |
| Urea Nitrogen(mg/dL) | 4 | 224 | 40 | 2.8 |
| Weight (Kg) | 28 | 277 | 37 | 2.59 |
| SpO_2_(%) | 0 | 100 | 14 | 0.98 |
| Respiratory Rate(breaths/min) | 8 | 75 | 2 | 0.14 |
| Age (year) | 19 | 96 | 0 | 0 |
| gender |  |  | 0 | 0 |
| Heart Rate(beats/min) | 20 | 182 | 0 | 0 |
| SOFA | 0 | 21 | 0 | 0 |
| SIRS | 0 | 4 | 0 | 0 |
| SAPS II | 8 | 114 | 0 | 0 |
| OASIS | 10 | 71 | 0 | 0 |

LDH: Lactate Dehydrogenase; SOFA: Sequential Organ Failure Assessment; APS III: Acute Physiology Score III; SIRS: Systemic Inflammatory Response Syndrome; SAPS II: Simplified Acute Physiology Score II; OASIS: Oxford Acute Severity of Illness Score; AKI: acute kidney injury.

**Supplementary Table 2.** **Perform multivariable logistic regression analysis to determine independent predictors that are associated with the development of AKI in IHCA patients.**

| **Variable** | **AOR*^1^*(95% CI*^1^*)** | **p-value** | |
| --- | --- | --- | --- |
| **Weight (Kg)** | 1.017(1.010-1.025) | | **<0.001** |
| **SpO_2_**(%) | 1.044(1.026-1.064) | | **<0.001** |
| **Sodium(mEq/L)** | 0.951(0.922-0.979) | | **<0.001** |
| Creatinine(mg/dL) | 1.058(0.961-1.186) | | 0.265 |
| **SOFA** | 1.107(1.046-1.172) | | **<0.001** |
| APS Ⅲ | 1.001(0.992-1.010) | | 0.836 |
| SAPS II | 1.009(0.994-1.025) | | 0.233 |
| **OASIS** | 1.075(1.049-1.102) | | **<0.001** |

*^1^*OR=Odds Ratio, CI=Confidence Interval; *^2^*False discovery rate correction for multiple testing SOFA: Sequential Organ Failure Assessment; APS III: Acute Physiology Score III; SIRS: Systemic Inflammatory Response Syndrome; SAPS II: Simplified Acute Physiology Score II; OASIS: Oxford Acute Severity of Illness Score; AKI: acute kidney injury; ICU: Intensive Care Unit; IHCA: in-hospital cardiac arrest.

Supplementary Table2 presented the results of a multivariable logistic regression analysis, which was a statistical method used to identify independent predictors associated with the occurrence of Acute Kidney Injury (AKI) among patients who had experienced Cardiac Arrest (CA). The analysis controlled for multiple variables simultaneously to determine their individual effects on the likelihood of AKI occurrence.


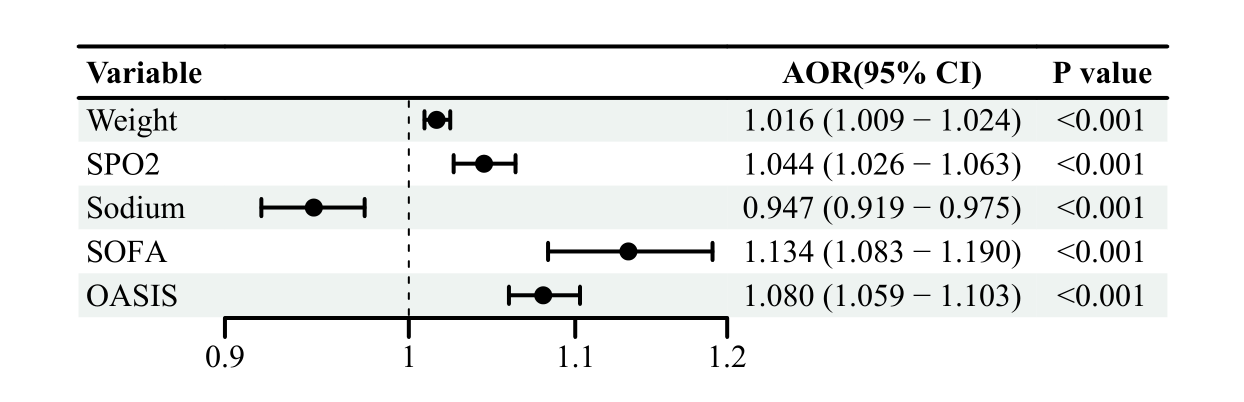


Supplementary Fig. 1. Forest graph to determine the probability of CA-AKI. The respective measurement units for each variable in the figure are as follows: weight (Kg), SpO_2_(%), Sodium (mEq/L). SOFA: Sequential Organ Failure Assessment; OASIS: Oxford Acute Severity of Illness Score; AKI: acute kidney injury; CA: cardiac arrest.

The simplified MDRD formula:

eGFR=186×(Scr)-1.154×(age)-0.203× (0.742 female) (Formula 4)

eGFR: estimated glomerular filtration rate (mL/min/1.73 m²); Scr: serum creatinine (mg/dL)
